# Another Journal

**Published:** 1881-07

**Authors:** 


*Another Journal.—The Herald of Dentistry* is a candidate for a place among the Dental Journals. It is edited and owned by Thomas O. Oliver, M. D., and published quarterly at 62

West 45th Street, New York City. Dr. Oliver, in making his bow "hopes and expects" that his journal will receive the support of the profession to such an extent that the present quarterly will soon be supplanted by a monthly and it in turn by a weekly. As Dr. O. is a "brand new" editor he will be pardoned for his enthusiasm, not only as to his hopes regarding a weekly journal, but for his promise that "it shall reach every practitioner of dentistry who reads the English language." He is sturdy in promising that it "shall not be the organ of any ring or clique; nor a college organ, neither a local society organ, nor yet a dental depot organ." Thus cutting himself off from all these helps, he seems to aim, at least in this initial number, at making it strictly an "Oliver" organ.

The "leader" in this number is devoted to an annihilation of Dr. J. Foster Flagg and "his book" We have no special disposition to defend Dr. Flagg, who can doubtless take care of himself and his book, but the animus of this attack by Dr. Oliver is so obvions as to attract attention. This is the style in which Dr. Oliver "goes for" Dr. Flagg:

"The dental profession are most probably possessed of sufficient intelligence to enable them to judge for themselves, profit by experience, and, at this enlightened age, are not likely to give very serious consideration to the commands of a self-anointed autocrat whose sublimated wisdom is of such a nature as to impress no one except a few satellites who know not why they follow him."

This is certainly a "new departure" in journalism, and has no equal since the some time remarkable productions of the editor of the *Canada Dental Journal.*

*The Herald of Dentistry* contains some good things, and some which will set people to thinking perhaps, at least those who are planning to "go abroad" and make a fortune. Those young gentlemen who feel that the struggles of genius in obtaining eminence at home in a profession such as ours are too arduous for them, will do well to read the letters from London and Paris, before they finally conclude to locate in England or Europe.

